# Epigenetic Regulations of Inflammatory Cyclooxygenase-Derived Prostanoids: Molecular Basis and Pathophysiological Consequences

**DOI:** 10.1155/2015/841097

**Published:** 2015-04-06

**Authors:** Hedi Harizi

**Affiliations:** Faculty of Dental Medicine, University of Monastir, 5000 Monastir, Tunisia

## Abstract

The potential relevance of prostanoid signaling in immunity and immunological disorders, or disease susceptibility and individual variations in drug responses, is an important area for investigation. The deregulation of Cyclooxygenase- (COX-) derived prostanoids has been reported in several immunoinflammatory disorders such as asthma, rheumatoid arthritis, cancer, and autoimmune diseases. In addition to the environmental factors and the genetic background to diseases, epigenetic mechanisms involved in the fine regulation of prostanoid biosynthesis and/or receptor signaling appeared to be an additional level of complexity in the understanding of prostanoid biology and crucial in controlling the different components of the COX pathways. Epigenetic alterations targeting inflammatory components of prostanoid biosynthesis and signaling pathways may be important in the process of neoplasia, depending on the tissue microenvironment and target genes. Here, we focused on the epigenetic modifications of inflammatory prostanoids in physiological immune response and immunological disorders. We described how major prostanoids and their receptors can be functionally regulated epigenetically and consequently the impact of these processes in the pathogenesis inflammatory diseases and the development of therapeutic approaches that may have important clinical applications.

## 1. Introduction

The proinflammatory environment induced by prostanoids is increasingly being recognized as a critical element for both inflammatory diseases and cancer [1]. The molecular and cellular basis of the immune regulation by prostanoids in physiological and pathological situations remain a topic of great interest. Biosynthesis of arachidonic-derived metabolites and receptors for major prostanoids are widely expressed throughout the immune system [2] and function at multiple levels in immune and inflammatory regulations [3]. Many cellular functions that are critical in the pathological processes such as carcinogenesis chronic inflammation pathologies and asthma are regulated by various prostanoids that are metabolites of Cyclooxygenase (COX) pathways, especially prostaglandin E2 (PGE2) [4]. This inflammatory bioactive lipid mediator is the best known and most well studied COX metabolite [5]. It has been reported that the endogenously released PGE2, the major metabolite of the COX pathway, suppresses multiple immune functions acting on most types of immune cells [6]. Among COX-derived prostanoids, PGE2 is one of the best characterized in terms of immunomodulation. It is a very attractive molecule in that it by itself exhibits both pro- and anti-inflammatory effects, particularly on dendritic cells (DCs). For example, in physiological conditions, PGE2 critically regulates the inflammatory phenotype and function of DCs [7], the most potent antigen-presenting cells (APC) of the immune system and known by their ability to stimulate naive, memory, and effector T cells [8]. COX-2-derived prostanoids are also involved in regulating various aspects of the T cell biology, including proliferation, apoptosis, cytokine secretion, differentiation, and chemotaxis [1, 9, 10].

In pathological conditions, overexpression of COX enzymes and abnormal production of COX-derived PGE2 [11, 12] have been reported to be linked to all carcinogenesis stages ranging from initiation to tumor progression [13]. Growing bodies of evidence have shown that COX-2-issued PGE2 markedly affects tumor angiogenesis [14–16]. When overexpressed, COX-2-synthesized PGE2 acts as a tumor promoter, regulates tumor angiogenesis [14], and potently alters the phenotype and function of circulating and tumor infiltrating cells, resulting in cancer-associated immunodeficiency [17]. Moreover, many tumors are associated with high levels of immunosuppressive PGE2 and an impaired differentiation and antigen-presenting function of DCs with an immature phenotype [18, 19]. In cancer, COX-2-derived PGE2 has also been reported to play crucial roles in the immunosuppressive function of Treg cells [20]. For these reasons and because of its inducible property, COX-2 expression must be tightly regulated and must be subjected to fine regulations.

COX-2 expression and PGE2 production can be induced by several inflammatory stimuli including growth factors and cytokines [21]. Proinflammatory cytokines, such as TNF-*α* [22, 23], IL-1*β* [24], and IFN-*γ* [25], have the potential to induce* COX-2* gene expression, whereas anti-inflammatory cytokines, in particular IL-4 [26], IL-13 [27, 28], and IL-10 [29], can inhibit* COX-2* gene induction and prostanoid biosynthesis. In addition to the environmental factors and the genetic background to inflammation, epigenetic acetylation of histone and nonhistone proteins by histone acetyltransferases plays a pivotal role in the expression of the proinflammatory COX-2/PGE2/EP receptor axis and its downstream signalling pathways.

## 2. Prostanoid Biosynthesis and Signaling Pathways

Prostanoids are inflammatory lipid signaling molecules synthesized by COX enzymes from phospholipase A2-released arachidonic acid, a 20 carbon polyunsaturated fatty acid present in most mammalian cell membranes and a major component of animal fats. Arachidonic acid is released by phospholipase A2 from the cell membrane and is converted to PGG2 and then reduced to PGH2 by COX enzymes. Two isoforms of COX enzyme are involved in the biosynthesis of prostanoids, COX-1 (also known as Prostaglandin-endoperoxide synthase, PTGS1) and COX-2 (also known as PTGS2). COX-2 is a membrane-bound and heme-containing enzyme which is a member of the mammalian heme-dependent peroxidase family. Although the expression profiles of both isoforms varies from tissue to tissue, COX-1, a housekeeping gene, is generally considered the constitutive form, being responsible for the homeostatic production of prostanoids and highly expressed in most tissues, including platelets, lung, prostate, brain, gastrointestinal tract, kidney, liver, and spleen [30]. However, COX-2, often referred to as the inducible or rate-limiting isoform, is usually undetectable in most normal tissues, being responsible for most of the prostanoid production during inflammation and markedly upregulated in various types of cancer, as well as in other diseases [1, 17, 31].* COX-2* gene can also be constitutively expressed in some tissues, such as the endothelium, kidney, gastrointestinal mucosa, and brain [32–34], and the constitutive expression of* COX-2* gene may be a contributing factor promoting tumoral pathologies, such as colorectal cancer [35].

Despite their different physiological functions, COX-1 and COX-2 enzymes are known by their different susceptibilities to inhibition by nonsteroidal anti-inflammatory drugs (NSAIDs) used for symptomatic treatment of inflammatory diseases, particularly due to their potent analgesic effect [36]. The common mechanism by which NSAIDs mediate their action is the inhibition of COX activity. Because COX-2 is thought to be the predominant isoform involved in the inflammatory response [37], most of the new research on anti-inflammatory drugs has been aimed at targeting the COX-2-inducible enzyme. Newly developed drugs that have high selectivity against COX-2, such as celecoxib, have been proved to be potent anti-inflammatory compounds without causing gastric toxicity.

PGE2 exerts its diverse and often antagonistic effects on target cells by various designated EP receptors (EP1–4) differentially expressed on many cell types and have been shown to differ in their signal transduction pathways [2, 38]. The use of agonists inducing changes in the levels of second messenger including, cAMP and free Ca^2+^ and the identification of G protein coupling by a variety of methods have allowed studying the signal transduction pathways of PGE2 receptors. The four EP receptors showed differential patterns of tissue distribution. EP1 mRNA is ubiquitously expressed in murine tissues, while high levels of EP3 receptor mRNA are found in adipose tissues, pancreas, kidney, and vena cava. EP4 mRNA is mainly expressed in the gastrointestinal tract, uterus, hematopoietic tissues, and skin, whereas EP2 receptor mRNA was found to be least abundant among EP receptors, with the highest expression occurring in the airways, ovary, bone marrow, and olfactory epithelium [39]. In addition to their differential patterns of tissue distribution, EP receptors have been shown to differ in their signal transduction pathways [40]. The EP1 receptor activates phospholipase C and phosphatidylinositol turnover and stimulates the release of intracellular calcium. The EP2 and EP4 receptors signal by stimulating adenylate cyclase, which increases the intracellular levels of cAMP. Signaling by the EP3 receptor is more complex because of multiple EP3 receptor isoforms generated by alternative splicing from a single EP3 gene. Because of these various signaling pathways, PGE2 can exert different and sometimes opposite effects on target cells [41]. Among the four PGE2 receptors, EP2 and EP4 mediate most, if not all, of the PGE2 effects on immune regulation [42–45].

Although, predominantly produced by APC, including DCs and macrophages [46, 47], PGE2 is known by its ubiquitous function because of the expression of various PGE2 EP receptors subtypes on immune and nonimmune cells [2, 7, 48]. In addition to environmental factors and genetic background to inflammation and immune disorders, prostanoid biosynthetic pathways and signaling can be regulated by epigenetic mechanisms.

## 3. Epigenetic Modifications of* COX* Genes

Many advances in our understanding of chromatin structure, histone modification, transcriptional activity, gene silencing, and DNA methylation have resulted in an increasingly integrated view of epigenetics and its impact in normal and pathological physiology. Inflammatory genes of the prostanoid pathways can be functionally regulated epigenetically. Dynamic epigenetic changes of prostanoid pathways can affect the biosynthesis of COX metabolites and/or the EP receptor signaling. Several epigenetic mechanisms, including DNA methylation, modification of histones, and noncoding RNAs [49, 50] are involved in these processes. It has been reported that histone acetylation is an important modification affecting gene transcription and is controlled by the action of histone acetyltransferase and deacetylase [51].

In mammals, changes in DNA methylation status and alterations in chromatin structure by histone modification represent the major epigenetic mechanisms involved in the regulation of gene transcription [52–54]. In fact, mammal's DNA is subjected to covalent modification that alters the chemical information content displayed in the major groove. This molecular process is classically associated with functions in genome defense and in genomic imprinting [55]. Described for the first time in calf thymus [56], methylation of cytosine is the most abundant DNA modification and occurs mainly during cellular differentiation [57]. DNA methylation is catalyzed by a well characterized family of enzymes, termed DNA methyltransferases [58]. In human cells, 60–80% of all CpG sites are highly methylated with some variation by cell type [59, 60].

Prostanoids are biologically active lipid mediators involved in several biological processes such as pain, fever, regulation of vascular tone, renal function, mucosal integrity, inflammation, angiogenesis apoptosis, and tumor growth. They are very attractive signaling molecules known by their critical role in regulating immune cell function [4, 7, 61]. These numerous biological processes are regulated by prostanoids through acting on secretion of various proteins, proteolysis, transcriptional activation, and epigenetic control. The biology of prostanoids has been extensively studied in cancer, and COX-2 pathway has emerged as a potential therapeutic target in some tumors [62, 63].

Genes encoding for inflammatory prostanoids and their receptors are subjected to epigenetic modifications by acetylation of core histone. Substantial evidences reported that* COX-2* is an important epigenetically controlled gene inflammatory gene in many pathological processes [63, 64]. Immune disorders, such as asthma and cancer, are characterized by the expression of various inflammatory genes that can be epigenetically regulated by acetylation of core histone. For example, increased activity of histone acetyltransferase and reduced activity of histone deacetylase have been observed in asthmatic patients [65]. The increased activity of histone acetyltransferase and the decreased activity of histone deacetylase resulted in the induction of inflammatory gene expression [65, 66]. Corticosteroids used as antiasthmatic treatment can exert their anti-inflammatory effects by regulating histone acetyltransferase or histone deacetylase activity [67]. Histone deacetylases are histone modifying enzymes identified as critical regulators of proinflammatory cascade, especially by acting on NF-*κ*B signaling pathway [68]. Recent study reported that epigenetic regulation is an important mechanism by which iloprost, a PGI2 analog, modulates asthma-related chemokines expression in monocytes [69]. Used as a potential candidate for treating asthma, PGI2 analog acts as an anti-inflammatory molecule inhibiting TNF*α* expression* via* MAPK signaling pathway and the downregulation of histone H3K4 trimethylation [70].

*COX-2* is also an epigenetically controlled gene in various cellular processes including the development, differentiation, and function of many immune cells, such as T regulatory (Treg) cells involved in the cancer-mediated immune suppression, and play a key role in immune regulations [71, 72]. Tumor-infiltrating Treg cells are able to suppress tumor-specific T cell immunity and contribute to the growth of tumors in a COX-2-dependent manner in mouse and human [64, 73, 74]. In cancer, COX-2-derived PGE2 plays essential roles in the immunosuppressive function of Treg cells in an autocrine and paracrine manner. These immunosuppressive cells express COX-2 and produce PGE2 upon differentiation [74]. The endogenously produced PGE2 released in the tumor microenvironment can inhibit T cell-mediated antitumor responses [75–77] and enhance the suppressive capacity of CD4+ CD25+ Treg cells [78]. These immunosuppressive cells are characterized by their specific molecular marker Foxp3 (Forkhead box protein P3), which is also an epigenetically modulated transcription factor involved in the development, differentiation, and function of Treg cells [79, 80]. Deficiency or spontaneous mutations of* Foxp3* gene leads to fatal autoimmune lymphoproliferation and autoimmune diseases caused by inactive Treg cells. In lung cancer, Foxp3 expression and Treg cell activity can be induced by COX-2-derived PGE2, and these effects can be reversed by COX-2 inhibitors and PGE2 receptor antagonists [81]. Taken together, these data suggested that targeting COX-2/PGE2 axis may have crucial roles in immune regulations in cancer microenvironment.

In addition to its indirect action, COX-2-derived PGE2 can exert direct effects on tumor cells. Several lines of evidence reported that PGE2 exhibits antiapoptotic and invasion-promoting effects thus supporting protumoral roles in some cancers [82, 83]. Evidence from epidemiological studies and clinical trials clearly indicates that aspirin, which is one of the most widely used drugs in the world, can protect against different types of cancer [84]. The COX-dependent mechanisms for the antitumoral effects of low dose aspirin have been clearly demonstrated especially in colorectal cancer [85] and the possible contribution of individual genetic cancer susceptibility to aspirin response [86] should be taken into consideration as an additional level of complexity in the understanding of the antitumor effects aspirin. Despite the individual genetic cancer susceptibility to aspirin, epigenetic control of some target genes appeared to play a crucial role in the antiproliferation of salicylates and NSAIDs. Accumulating data reported that targeting the epigenome may play a key role in cancer chemoprevention [87, 88].

Since the cancer genome usually contains both hyper- and hypomethylated genes to increase invasion, proliferation, and metastasis, it is not surprising that* COX-2* gene can be upregulated or downregulated by epigenetic mechanisms. Several lines of evidence reported that COX-2 expression and prostanoid biosynthesis may be affected by various perturbations that are present in most cancer types. For example, silencing of* COX-2* gene by hypermethylation has been reported in human colon cancer [89, 90]. Many data from epidemiological and clinical studies reported that COX-2 expression and prostanoid biosynthesis are frequently overexpressed in many cancer types [11, 91, 92]. The general consensus is that the elevated expression of COX-2 protein is commonly observed in many chronic inflammatory diseases and cancer [93]. However, other studies clearly reported that COX-2 protein may be downregulated in tumors of a subset of colorectal and gastric cancer patients, and the downregulation of the* COX-2* gene appeared to be strongly correlated with CpG island DNA methylation [90, 94]. These conflicting data suggested that other factors may contribute to the regulation of* COX-2* gene in chronic inflammatory diseases and cancer. Kikuchi et al., [95] reported that the downregulation of the* COX-2* gene is mediated by histone deacetylation in gastric cancer, whereas* COX-1* gene has only been found epigenetically silenced by promoter hypermethylation in pancreatic tumors [96]. The hypermethylation of* COX-2* gene by histone deacetylation has been described in several tumor types such as gastric [95], pancreatic [96], breast [97, 98], hepatic [99–101], nasopharyngeal [102], prostate [103, 104], esophageal [105, 106], and cervical tumors [107, 108]. Collectively, these data clearly showed that epigenetic alterations play a critical role in the deregulation and aberrant expression of the genes of the COX pathway in different types of cancer.

PGE2 regulates numerous biological processes by modulating transcriptional activation, proteolysis, secretion of various proteins, and epigenetic control. This lipid mediator could not only be regulated epigenetically but also act by itself as a potential epigenetic regulator of the transcriptional mechanisms of other inflammatory processes. In fact, given its increased production in chronic inflammatory diseases and cancer, it is not surprising that PGE2 could act as a potential regulator of other inflammatory genes. For example, it has been reported that PGE2 activates* IL-8 *transcription gene through specific demethylation of the CpG site and abnormal acetylation of histone H3 in the* IL-8 *promoter gene in human astrocytoma [109].

## 4. Epigenetic Modifications Targeting EP Receptor Signaling 

PGE2 exerts its effects on target cells by binding on four EP receptors with seven transmembrane domains to trigger various intracellular signal transduction cascades, impacting cell growth, survival, apoptosis, and immune responsiveness [110]. The existence of four subtypes of PGE2 receptors is remarkable given that each of the other prostanoids each has only a single receptor. This complex family of EP receptors coupled to distinct intracellular signals provides a molecular basis for the diverse physiological and sometimes opposing actions of PGE2 [111]. Differential expression of these EP receptors mediates the diverse and often antagonistic effects of PGE2 on a variety of cell types [37]. Like many inflammatory signaling molecules, genes encoding for all of the PGE2 receptors are subjected to fine regulations by various mechanisms. Growing bodies of evidence indicate that epigenetic modifications play a critical role in the deregulation of the genes of the EP receptors with critical consequences on downstream signaling [112, 113]. DNA CpG methylation and histone posttranslational modifications have been reported to be major epigenetic mechanisms affecting EP receptor expression and synthesis in most cancer types [114]. It has been reported that histone acetylation was found to be a critical regulator of EP receptor expression in cancer [113]. For example, EP receptor expression appeared to be frequently dysregulated in non-small lung cancer (NSLC), via DNA CpG methylation and histone posttranslational modifications [115]. The EP1 receptor signaling, which is involved in PGE2-mediated activation of MAPK/ERK-induced cellular proliferation [116], was found to be downregulated by histone deacetylase inhibitors in NSCLC cell lines. The EP2 expression appeared to be predominantly downregulated by DNA CpG methylation and may have an important role in the pathogenesis of NSLC [113]. The epigenetic downregulation of the* EP2 *gene by DNA CpG methylation was also observed to be associated with progression of neuroblastomas [117]. In addition,* EP2* gene has been found to be hypermethylated in lung [113] and gastric [118, 119] cancers. In idiopathic pulmonary fibrosis, DNA hypermethylation appeared to be responsible for diminished EP2 expression levels and PGE2 resistance [120]. The use of DNA methylation inhibitors 5-aza-2′-deoxycytidine and zebularine as well as DNA methyltransferase-specific siRNA increased EP2 mRNA and protein expression levels and restored PGE2 responsiveness in fibrotic fibroblasts. Shoji et al. [112] have reported that the PGE2 receptor subtype EP3 plays a critical role in suppression of cell growth and that its downregulation enhances colon carcinogenesis at a later stage. DNA methylation of the EP3 receptor gene has been demonstrated in both colon cancer and oesophageal cancer and the use of a DNA demethylating agent restored EP3 receptor expression in various cell lines [112, 121]. Hypermethylated* EP3 *[105] and* EP4 *[118] genes have been reported in colorectal and gastric tumors, respectively. Moreover, it has been demonstrated that DNA methyltransferase inhibitors and histone deacetylase inhibitors upregulate EP3 and EP4 receptor expression in cancer lung [113]. Collectively, these data clearly demonstrate that EP receptor signaling can be functionally regulated epigenetically and may represent a potential targets for epigenetic therapies in the treatment of inflammatory disorders, such as cancer, autoimmune diseases, and asthma.

## 5. Concluding Remarks

The inflammatory COX-derived prostanoids are increasingly being recognized as critical regulators for both immunity and immunological diseases. Substantial data clearly showed that prostanoid biosynthesis and signaling pathways are altered in these pathological processes. Pharmacological targeting of prostanoid biosynthesis and/or signaling with NSAIDs, COX-2 selective inhibitors, and EP receptor antagonists has been investigated for many years with promising results at both preventive and therapeutic levels. There are many fascinating accumulating data on the biology of inflammatory prostanoids in normal and pathological settings. Numerous studies reported that epigenetic control is an important mechanism by which COX/PGE2/EP receptor axis can be markedly modulated. Epigenetic modifications emerged as additional levels of complexity to the understanding of the regulation and functionalities of the COX pathway and prostanoid signaling in physiological and pathological settings. Investigating the epigenetic alterations that impact both COX enzymes and prostanoid receptors may inform on pathological events that characterize the immunomodulatory functions of these inflammatory lipid mediators in various immune disorders and may be relevant for the efficient clinical use of NSAIDs, COX-2 selective inhibitors, and EP receptor antagonists at both preventive and therapeutic levels. Thus, besides NSAIDs and EP receptor antagonists, epigenetic therapies, including DNA methyltransferase inhibitors and histone deacetylase inhibitors, should be considered in studying cellular proliferation and invasiveness in immune disorders and in the development of therapeutic approaches aimed at targeting inflammatory prostanoid biosynthesis and/or signaling. Epigenetic regulations, especially alterations in DNA methylation, are intimately linked to the development of various human tumors. Because the cancer genome usually contains both hyper- and hypomethylated genes to increase invasion, proliferation, and metastasis, epigenetic control of inflammatory genes such as* COX-2* should be taken into consideration as a potential therapeutic target in tumoral pathology. Thus, the epigenetic regulation of prostanoids is extremely interesting and deserves further investigations.
